# Room of Horrors: Einfluss auf die Haltung zur Bewohner*innensicherheit – Eine Prä-post-Studie

**DOI:** 10.1007/s00391-025-02473-x

**Published:** 2025-07-31

**Authors:** Graciana-Virginye Versteeg, Theresa A. Forbrig, Johannes Gräske

**Affiliations:** 1 Fachbereich II, Alice Salomon Hochschule Berlin, Alice-Salomon-Platz 5, 12627 Berlin, Deutschland; 2 Pflegewohnzentrum Kaulsdorf-Nord gGmbH, Berlin, Deutschland; 3 Charité Universitätsmedizin Berlin, Berlin, Deutschland

**Keywords:** Simulationsbasiertes Lernen, Fort- und Weiterbildung, Patientensicherheit, Fehlerkultur, Qualitätsmanagement, Simulation based learning, Professional development, Patient safety, Safety culture, Quality management

## Abstract

**Hintergrund:**

Sicherheitsrisiken in der stationären Langzeitpflege entstehen durch komplexe Pflegesituationen und die Vulnerabilität der Bewohner*innen. Simulationsbasierte Methoden wie die Room-of-Horrors(RoH)-Intervention können das Bewusstsein der Mitarbeitenden für solche Risiken stärken. Übergeordnetes Ziel der Arbeit war es zu untersuchen, wie sich eine RoH-Intervention auf die Haltung der Mitarbeitenden bezüglich der Bewohner*innensicherheit in der Langzeitpflege auswirkt.

**Methodik:**

Eine schriftlich standardisierte Prä-post-Erhebung mit 176 Teilnehmenden erfasste die Haltung gegenüber Sicherheitsrisiken mithilfe des „German short Version of the Attitudes to Patient Safety Questionnaire“ (G-APSQshort).

**Ergebnisse:**

Der G‑APSQshort-Score stieg signifikant von 5,4 auf 5,6 Punkte (*p* < 0,001). Besonders Betreuungskräfte profitierten von der Intervention (*p* < 0,001). Von allen Teilnehmenden würden 77,3 % das RoH-Konzept weiterempfehlen, und 80,2 % der Teilnehmenden wünschen sich regelmäßige RoH-Interventionen.

**Diskussion:**

Die RoH-Intervention verbessert die Haltung der Mitarbeitenden bezüglich Bewohner*innensicherheit, wobei die praktische Relevanz weiterevaluiert werden sollte. Die RoH-Methode fördert die Sicherheitskultur und verbessert die Pflegequalität in der Langzeitpflege.

## Abstract (en)

**Background:**

Safety risks in residential long-term care arise from complex care situations and the vulnerability of residents. Simulation-based methods, such as a room of horrors (RoH) intervention, can enhance staff awareness of these risks. This study aimed to investigate how staff attitudes toward resident safety in long-term care develop in the context of a RoH intervention.

**Method:**

A written standardized pre-post survey with 176 participants was conducted, measuring attitudes toward safety risks using the German short version of the attitudes to patient safety questionnaire (G-APSQshort).

**Results:**

The G‑APSQshort score significantly increased from 5.4 to 5.6 points (*p* < 0.001), with care assistants showing the greatest improvement (*p* < 0.001). Additionally, 77.3% of participants would recommend the RoH concept further and 80.2% expressed a desire for regular RoH interventions.

**Conclusion:**

The RoH intervention enhances staff awareness and attitudes toward resident safety, fostering a safety culture and improving care quality in long-term care; however, the practical relevance of the findings requires further evaluation.

Die zunehmenden Anforderungen an individuelle, lebensweltnahe Versorgungssettings führt zu besonderen Herausforderungen hinsichtlich der Sicherheit für die Bewohner*innen [1]. Die Weltgesundheitsorganisation (WHO) definiert Patient*innensicherheit als organisierte Maßnahmen im Gesundheitswesen, die durch Prozesse, Verhaltensweisen, Technologien und Umgebungen Risiken nachhaltig senken, vermeidbare Schäden reduzieren, Fehler unwahrscheinlicher machen und deren Auswirkungen minimieren. Dennoch treten Ereignisse wie Sturz [1], Medikationsfehler [2] oder Dekubitus [3] wiederholt auf. Mehr als die Hälfte der unerwünschten Ereignisse im Kontext der Pflege gelten als vermeidbar, was auch für rund 40 % der Krankenhauseinweisungen aus Pflegeeinrichtungen gilt [4].

Der Begriff Patient*innensicherheit ist international gebräuchlich, bezieht sich jedoch überwiegend auf den klinischen Kontext. In der stationären Langzeitpflege ist der Begriff Patient*in nur bedingt passend, da die betreuten Personen meist dauerhaft dort leben. Im Unterschied zu Krankenhauspatient*innen zeichnen sie sich durch längere Aufenthaltsdauer, höhere Vulnerabilität, Multimorbidität und eingeschränkte Selbstbestimmung aus. Daher wird in der vorliegenden Arbeit von Bewohner*innensicherheit gesprochen. Grundlage für eine positive Fehlerkultur ist eine veränderte Haltung bzw. Einstellung zur Bewohner*innensicherheit [5], dazu gehört beispielsweise die kritische Selbstreflexion oder praktische Handlungsbereitschaft. Langzeitpflegeeinrichtungen sind angehalten, entsprechende Interventionen, die langfristig das Risiko für Bewohnende verringern, einzuführen.

Eine Möglichkeit ist der Room of Horrors (RoH). Ein RoH stellt einen innovativen, interaktiven, kostengünstigen und leicht zugänglichen Trainingsraum dar, in dem Mitarbeitende real erlebbare Szenarien durchlaufen. Das Konzept wird schon länger im pflegerischen Kontext angewendet [6]. In diesen Szenarien sind installierte Fehler und Risiken zu einem spezifischen Thema verborgen; diese müssen von den Teilnehmenden erkannt werden [7, 8]. Erste Evaluationen zeigen positive Effekte für die Aufmerksamkeit über einen Zeitraum von einem Monat [9]. In ihrer systematischen Übersichtsarbeit geben Lee et al. Fehlererkennungsraten zwischen 24,5 und 90,1 % an [6]. Die Durchführung eines RoH geht u. a. mit dem Ziel einher, das Bewusstsein der Mitarbeitenden zu spezifischen sicherheitsrelevanten Themenfeldern zu schärfen [8]. Des Weiteren zielt ein RoH darauf ab, die Fähigkeit zur Erkennung, zum Verständnis und zur angemessenen Reaktion auf potenzielle Risiken und Gefahren im beruflichen Kontext zu fördern. Dies erfolgt durch die Schaffung realitätsnaher Erfahrungen [8]. Des Weiteren werden die Fähigkeit der Teilnehmenden zum kritischen Denken, ihre Beobachtungsfähigkeit, ihr Situationsbewusstsein sowie ihre Problemlösekompetenz gestärkt [7]. Unklar ist bislang, ob sich RoH dazu eignen, eine positive Haltung zum Thema Bewohner*innensicherheit zu steigern. Daher soll in diesem Beitrag die folgende Forschungsfrage beantwortet werden: Inwieweit verändert die RoH-Intervention die Haltung zur Bewohner*innensicherheit?

## Methodik

Die Intervention fand im Sommer 2024 bei einem Träger mit 5 Einrichtungen der (teil-)stationären Langzeitpflege statt. Es wurde ein quantitatives Prä-post-Design, das die Erfassung von Veränderungen hinsichtlich der Haltung gegenüber der Bewohner*innensicherheit vor und nach der RoH-Intervention ermöglichte, gewählt.

### Stichprobe

Die RoH-Intervention wurde als Fortbildung allen Mitarbeitenden der direkten Versorgung (*n* = 234), unabhängig von der Berufszugehörigkeit (beispielsweise Pflegefachperson, Alltagsbegleiter*innen (ohne formale Ausbildung, Betreuungskräfte (nach § 45b bzw. § 53 SGB IX))) angeboten. Ausgeschlossen wurden Lernende (Praktikant*innen, Auszubildende etc.). Ziel war es, eine möglichst heterogene Stichprobe sicherzustellen, um die Auswirkung der Intervention in interprofessionellen Teams zu analysieren. Alle Mitarbeitenden erhielten eine schriftliche Information und einen konkreten Terminvorschlag, der sich an den Dienstzeiten (direkt davor oder danach) orientierte. Die Teilnahmen an der Fortbildung und der Evaluation waren getrennt voneinander möglich. Eine Absage war nicht notwendig, lediglich das Nichterscheinen wurde für die Berechnung der Teilnahmequote dokumentiert. Die Gruppen setzten sich aus bestehenden Teams der Wohnbereiche („Realgruppen“) zusammen, um eine hohe Alltagsnähe zu sichern. Mindestens eine Pflegefachperson pro Gruppe stellte sicher, dass auch pflegefachliche Fehler erkannt werden konnten.

### Intervention

**Abb. 1:**
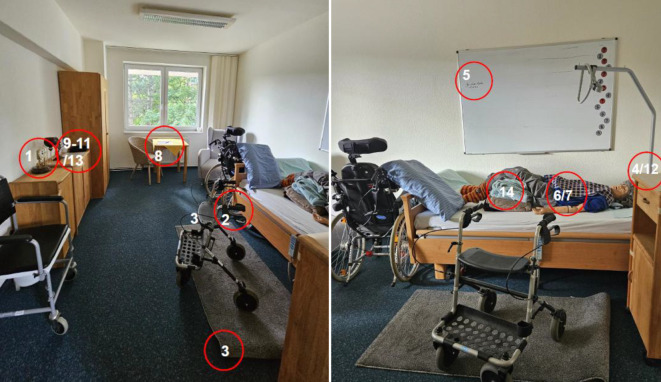
Room of Horrors, teilstationäre Langzeitpflege. *1* Uhr steht [Pflegeprozess], *2* Rollator: nicht angebremst [medizinisches Gerät], *3* Ecke des Läufers ist umgeklappt [Patient*innenunfälle], *4* Händedesinfektion steht in Reichweite des*der Bewohner*in, Flasche offen, nicht mit Anbruchsdatum versehen [nosokomiale Infektion], *5* falscher Name im Bewohner*innenzimmer [Pflegeprozess], *6* Pflaster in linker Ellenbeuge bei Pflasterallergie [Pflegeprozess], *7* Stoma verkehrt herum angebracht [Pflegeprozess], *8* keine Blutdruckkontrollen bei auffälligen Werten [Pflegeprozess], *9* Blutdruckgerät nicht funktionsfähig [medizinisches Gerät], *10* Präparat stimmt nicht mit Medikationsplan überein [Medikamentenmanagement + Dokumentation], *11* Stempel der medizinischen Praxis fehlt, falsche Dosis Torasemid, Präparat ohne Namen des*der Bewohner*in [Medikamentenmanagement + Dokumentation], *12* Notrufklingel fehlt [Pflegeprozess], *13* Ernährungsprotokoll unvollständig, Milchreis bei Laktoseintoleranz verabreicht [Ernährungsmanagement + Dokumentation], *14* Schocklagerung bei Verdacht auf Apoplex [Pflegeprozess]

**Abb. 2:**
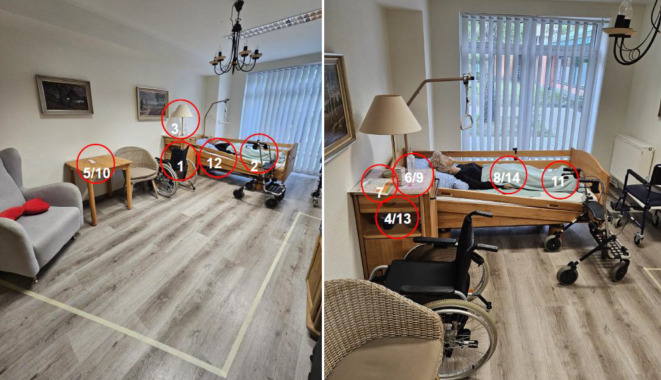
Room of Horrors, stationäre Langzeitpflege. *1* Rollstuhl: Bremse nicht feststellbar [medizinisches Gerät], *2* Rollator: Griffe verschieden hoch, Bremse nicht feststellbar [medizinisches Gerät], *3* Nachtlampe: Leuchtmittel defekt [Patient*innenunfälle + Gebäude/Einrichtung/Infrastruktur], *4* Hühnerei bei Hühnereiweißallergie [Ernährungsmanagement], *5* Metamizolallergie + verordnetes Novalgin [Medikamentenmanagement], *6* Novaminsulfontropfen gestellt [Medikamentenmanagement], *7* falsches Verbandmaterial [Pflegeprozess], *8* Inkontinenzmaterial verdreht, vorn/hinten vertauscht [Pflegeprozess], *9* NaCl-Lösung, 0,9 %ig, ohne Anbruchsdatum und mit Kanüle versehen [nosokomiale Infektion], *10* Wundbeschreibung fehlt [Dokumentation], *11* Kompressionsstrümpfe faltig, eine Seite linksrum angezogen [Pflegeprozess], *12* Bettseitenschutz hochgestellt [Pflegeprozess], *13* Becher Kaffee mit Schimmel bedeckt [nosokomiale Infektion], *14* Schutzkappe der Kanüle liegt unter Bewohner*in [Pflegeprozess]

Die RoH-Intervention bestand aus realitätsnahen Simulationen in einem nachgestellten Bewohner*innenzimmer. Sie findet in den üblichen 3 Schritten statt: Prebriefing, Intervention/Durchführung, Debriefing [10]. Das Debriefing sollte auf theoretischen Modellen basierend durchgeführt werden [11]. Um den Grad der Relevanz zu erhöhen, erfolgte die Szenarienentwicklung in Zusammenarbeit mit 6 Praxisanleitenden des Unternehmens. Bei der Erstellung der Szenarien wurde sich an den Vorfallstypen der WHO [5] orientiert. Dabei wurden solche, die kaum bis gar nicht im Bereich der (teil-)stationären Langzeitpflege vorzufinden sind, beispielsweise Blutprodukte, nicht in den Szenarien berücksichtigt. Folgende Bereiche wurden berücksichtigt: Patient*innenunfälle, Ernährungsmanagement, Medikamentenmanagement, klinische Prozesse, nosokomiale Infektionen, Dokumentation, Ressourcen/Organisationsmanagement und medizinische Geräte. Es wurde ein Szenario für die teilstationäre (Abb. 1) und ein Szenario für die stationäre Langzeitpflege (Abb. 2) entwickelt. Bei der Struktur der Szenarien wurde sich an den Szenarien der Patientensicherheit Schweiz [12] orientiert. Die Teilnehmenden hatten die Aufgabe, diese Fehler zu identifizieren und deren potenziellen Auswirkungen einzuschätzen. Die Anzahl der Fehler orientiert sich an denen vergleichbarer Studien und betrug in beiden Szenarien jeweils *n* = 14. Beide RoH wurden einem Prätest unterzogen, es gab keine Änderungen im Anschluss. Über den kompletten Interventionszeitraum blieb der RoH unverändert, sodass für alle Gruppen die gleichen Bedingungen galten. Im Anschluss an die RoH-Intervention gab es ein standardisiertes Debriefing, um die Vergleichbarkeit zwischen den Gruppen sicherzustellen. Das Debriefing orientierte sich an der 3‑D-Methode (Diffusing, Discovering, Deepening) von Ziegmont et al. [13], dauerte rund 45 min und fand in den jeweiligen Gruppen statt. Das Debriefing ist integraler Bestandteil der RoH-Simulation.

### Datenerhebung

Die Datenerhebung erfolgte mittels digitalem Fragebogen direkt vor dem Betreten des RoH und direkt nach dem Debriefing. Die Teilnehmenden konnten die Befragung jederzeit ohne Angabe von Gründen abbrechen. Um einen Prä-post-Vergleich zu ermöglichen, generierten die Teilnehmenden ein eigenes Pseudonym.

### Fragebogen

Zunächst wurden persönliche (beispielsweise Alter und Geschlecht) sowie berufliche (beispielsweise Berufserfahrung, Abschluss) Daten erfragt. Im Anschluss konnten die Teilnehmenden in der Postbefragung den RoH bewerten.

Die Haltung der Teilnehmenden gegenüber Sicherheitsrisiken wurde mithilfe des „German short version of the Attitudes to Patient Safety Questionnaire“ (G-APSQshort) gemessen [14]. Das Instrument wurde in Anlehnung an Kaveh et al. [15] sprachlich an den pflegerischen Kontext angepasst, so wurde z. B. der Begriff „Medizinstudium“ durch „Ausbildung“ ersetzt. Die Anpassungen wurden von dem Autor (J.G.) vorgenommen und anschließend von beiden Autor*innen (G.-V.V., T.A.F.) unabhängig voneinander validiert. Es gab keine Unstimmigkeiten. Der Fragebogen umfasst 14 Fragen (beispielsweise: „Ich wäre bereit dazu, jeden Fehler, den ein anderer begangen hat, zu melden. Ganz gleich, wie ernsthaft die Folgen für den*die Patienten*in gewesen sind.“) mit 6 Subskalen (beispielsweise Patient*innensicherheitstraining bis heute) und einem Gesamtpunktwert (Zusatzmaterial online). Alle Fragen wurden auf einer 7‑stufigen Likert-Skala (1 „Ich lehne stark ab“ bis 7 „Ich stimme stark zu“) beantwortet. Aus den jeweiligen Fragen einer Subskala bzw. des Gesamtpunktwerts wurde der Mittelwert gebildet, wobei höhere Punktwerte einer höheren Zustimmung entsprachen [14].

### Datenauswertung

Die Datenbeschreibung erfolgt mittels typischer Lage- und Streuungsmaße sowie absoluter und relativer Werte. Zur Untersuchung der Veränderung zwischen den Prä- und Postmessungen wurden gepaarte *t*-Tests durchgeführt. Zusätzlich wurden Korrelationen und eine Varianzanalyse (ANOVA) genutzt, um die Unterschiede zwischen Berufsgruppen zu ermitteln. Alle statistischen Analysen erfolgten mittels IBM SPSS (Version 29, IBM Corp., Armonk, NY, USA) zu einem Signifikanzniveau < 0,05.

### Datenschutz und Ethik

Die Teilnahme an der Intervention war freiwillig. Alle Teilnehmenden wurden schriftlich und mündlich über die Intervention aufgeklärt. Eine Nichtteilnahme führte zu keinen negativen Konsequenzen. Das Vorgehen basiert auf den ethischen Grundsätzen der Deklaration von Helsinki und dem Ethikkodex des International Council of Nurses.

## Ergebnisse

Insgesamt nahmen von 234 eingeladenen Mitarbeitenden 186 Personen an den 9 verschiedenen Terminen der RoH-Intervention teil. Dies entspricht einer Teilnahmequote von 79,5 %. Die Gruppengröße variierte zwischen 3 und 8 Personen und umfasste in den meisten Fällen Mitarbeitende aus dem Stammteam des jeweiligen Wohnbereichs. Vor der Datenauswertung wurden die ausgefüllten Fragebogen gesichtet und bereinigt. Von ursprünglich 186 Personen, welche die Befragung begonnen haben, konnten 176 Teilnehmende in die Auswertung einbezogen werden.

Die Teilnehmenden waren im Durchschnitt 48,8 (SD 11,0) Jahre alt und zu 90 % weiblich. Die Teilnehmenden identifizierten im Durchschnitt 8,2 (SD 2,6) von 14 installierten Fehler (58,6 %) (Tab. 1). Besonders häufig wurden Fehler aus den Kategorien „nosokomiale Infektionen“, „Medikamentenmanagement“, „Patient*innenunfälle“ und „klinische Prozesse“ erkannt. Schwierig zu identifizieren waren Fehler im Bereich „medizinische Geräte“ und „Ressourcen/Organisationsmanagement“. Zwischen der Gruppe, die die Postbefragung durchgeführt hat, und denen, die das nicht getan haben, bestehen keine signifikanten Unterschiede in den Charakteristika.

**Tab. 1:** Beschreibung der Stichprobe

	Gesamtstichprobe (*n* = 178)	Follow-up (*n* = 149)	Drop-out (*n* = 27)	*p*-Wert
*Alter*, MW (SD)	48,4 (11,0)	48,4 (11,1)	48,0 (10,6)	0,859
*Geschlecht, n* (%)				
Weiblich	160 (89,9)	134 (90,9)	26 (96,3)	0,472
Männlich	16 (9,0)	15 (10,1)	1 (3,7)	
*Berufsabschluss, n* (%)				
Pflegefachassistenz mit Ausbildung	46 (25,8)	44 (29,5)	2 (50)	0,247
Pflegefachperson	67 (37,6)	67 (45,0)	0 (0,0)	
Alltagsbegleiter	18 (10,1)	17 (11,4)	1 (25,0)	
Betreuungskraft	10 (5,6)	9 (6,0)	1 (25,0)	
Andere	12 (6,7)	12 (8,1)	0 (0,0)	
*Berufserfahrung*, MW (SD)	14,8 (10,4)	15,0 (10,6)	14,6 (9,1)	0,864
*Gruppengröße*	–			0,533
≤ 3	33 (18,5)	29 (19,5)	4 (14,8)	
4	44 (24,7)	37 (24,8)	7 (25,9)	
5	80 (44,9)	69 (46,3)	11 (40,7)	
≥ 6	19 (10,7)	14 (9,4)	5 (18,5)	
*Gefundene Fehler*, MW (SD)	8,2 (2,6)	8,2 (2,6)	8,0 (2,5)	0,658

Abweichungen zur angegebenen Gesamtzahl beruhen auf fehlenden Angaben

Vor der eigentlichen Intervention lagen die G‑APSQshort-Punktwerte im oberen Drittel. Nach der Intervention zeigen die Punktwerte eine Verbesserung der Haltung der Teilnehmenden gegenüber der Bewohner*innensicherheit in allen 6 Subskalen des G‑APSQshort sowie im Gesamtscore. Der größte signifikante Anstieg ist in der Subskala „Bewohner*innensicherheitstraining bis heute“ zu erkennen. Weitere signifikante Verbesserungen waren im Gesamtpunkwert und in den Subskalen „Arbeitszeit als Fehlerquelle“, „Unvermeidbarkeit von Fehlern“ und „Bedeutung von Bewohner*innensicherheit im Lehrplan“ (alle *p* < 0,05) zu erkennen (Tab. 2).

**Tab. 2:** Prä- und Postvergleich des G‑APSQshort

Subskalen	Gesamtwert, MW (SD) (*n* = 149)		Pearson (*n* = 149)		Gepaarter *t*-Test (*n* = 149)
	Prä	Post	r	*p*-Wert	*p*-Wert
Bewohner*innensicherheitstraining bis heute	5,3 (1,5)	5,8 (1,3)	*0,705*	*<* *0,001*	*<* *0,001*
Sicherheit, Fehler zu kommunizieren	5,5 (1,5)	5,6 (1,4)	*0,758*	*<* *0,001*	0,346
Arbeitszeiten als Fehlerquellen	5,4 (1,5)	5,6 (1,4)	*0,756*	*<* *0,001*	*0,011*
Unvermeidbarkeit von Fehlern	5,7 (1,4)	5,9 (1,3)	*0,583*	*<* *0,001*	*0,033*
Die Rolle der zu Pflegenden bei Fehlern	4,9 (1,7)	5,0 (1,6)	*0,719*	*<* *0,001*	0,342
Bedeutung von Bewohner*innensicherheit im Lehrplan	5,9 (1,3)	6,2 (1,2)	*0,720*	*<* *0,001*	*<* *0,001*
Gesamtpunktwert	5,4 (1,1)	5,6 (1,1)	*0,839*	*<* *0,001*	*<* *0,001*

*MW* arithmetisches Mittel, *SD* Standardabweichung

Die Intervention in der Subskala „Bewohner*innensicherheitstraining bis heute“ hat für Betreuungskräfte einen signifikant größeren Nutzen als für Pflegeassistent*innen (ANOVA b = −1,218, *p* < 0,001) und Pflegefachpersonen (ANOVA b = −1,368, *p* < 0,001; Tab. 3). Das Ergebnis zeigt sich ebenfalls im Gesamtpunktwert für die Pflegefachpersonen (b −0,463, *p* = 0,036).

**Tab. 3:** ANOVA der Subskalen des G‑APSQshort

***Unabhängige Variablen***	Bewohner*innensicherheitstraining bis heute		***Sicherheit, Fehler zu kommunizieren***		***Arbeitszeiten als Fehlerquelle***		***Unvermeidbarkeit von Fehlern***	
	***b***	***p‑Wert***	***b***	***p‑Wert***	***b***	***p‑Wert***	***b***	***p‑Wert***
Konstanter Term	0,717	0,372	−0,847	0,288	0,804	0,311	0,769	0,414
Geschlecht^*^								
Weiblich	−0,163	0,561	−0,121	0,663	−0,43	0,876	−0,408	0,215
Berufsabschluss^**^								
Pflegeassistent*in	*−1,218*	*<* *0,001*	0,266	0,472	−0,400	0,278	0,121	0,782
Pflegefachperson	*−1,368*	*<* *0,001*	0,039	0,915	−0,711	0,051	−0,127	0,768
Alltagsbegleiter*in	−0,604	0,170	0,112	0,796	−0,715	0,101	0,680	0,187
Andere	−0,674	0,144	−0,202	0,658	−0,485	0,287	−0,728	0,178
Gruppengröße^***^								
≤ 3	0,088	0,808	0,048	0,894	−0,161	0,652	−0,340	0,421
4	−0,243	0,472	0,330	0,325	−0,075	0,821	−0,453	0,253
5	0,133	0,669	0,477	0,124	−0,017	0,956	−0,122	0,737
Alter^§^	0,005	0,520	0,004	0,578	0,007	0,391	0,002	0,837
Anzahl gefundener Fehler^§^	*0,085*	*0,023*	0,050	0,176	−0,033	0,366	−0,006	0,896
***Unabhängige Variablen***	***Die Rolle der zu Pflegenden bei Fehlern***		Bedeutung von Bewohner*innensicherheit im Lehrplan				***Gesamtpunktwert***	
	***b***	***p‑Wert***	***b***		***p‑Wert***		***b***	***p‑Wert***
Konstanter Term	−0,556	0,555	0,448		0,548		0,214	0,656
Geschlecht^*^								
Weiblich	0,137	0,676	−0,061		0,814		−0,115	0,493
Berufsabschluss^**^								
Pflegeassistent*in	−0,720	0,101	−0,248		0,475		−0,377	0,092
Pflegefachperson	−0,099	0,818	−0,276		0,418		*−0,463*	*0,036*
Alltagsbegleiter*in	−0,777	0,132	−0,435		0,287		−0,272	0,301
Andere	0,113	0,833	−0,365		0,394		−0,398	0,149
Gruppengröße^***^								
≤ 3	0,005	0,990	−0,129		0,699		−0,097	0,652
4	−0,242	0,541	−0,523		0,097		−0,164	0,415
5	−0,17	0,963	0,026		0,929		0,089	0,631
Alter^§^	*0,020*	*0,035*	0,009		0,247		0,008	0,120
Anzahl gefundener Fehler^§^	−0,006	0,897	−0,015		0,672		0,019	0,394

Referenzkategorien: *männlich; **Betreuungskraft; ***≥ 6

^§^Kontinuierliche Co-Variable, kursivgedruckte Werte indizieren ein signifikantes Ergebnis zum 5 %-Niveau

77,3 % der Teilnehmenden würden die RoH-Intervention „sehr weiterempfehlen“, während 80,2 % regelmäßige RoH-Trainings in der Praxis befürworten.

### Diskussion

Das Ziel der vorliegenden Arbeit war es, den Einfluss einer RoH-Intervention auf die Haltung von Mitarbeitenden der stationären Langzeitpflege zur Bewohner*innensicherheit zu untersuchen. Der Anteil der identifizierten Fehler (58,6 %) lag leicht über der Studie von Zimmermann et al. im Krankenhaus (47 %; [10]). 27 Personen haben an der Postbefragung nicht teilgenommen. Eine Analyse der Merkmale dieser Teilnehmenden mit den Personen, die die Postbefragung durchgeführt haben, ergab keine signifikanten Unterschiede, sodass nicht von einer systematischen Verzerrung ausgegangen werden muss.

Vor der Intervention lagen die G‑APSQshort-Werte der Teilnehmenden über dem Niveau von Pflegestudierenden [16]. Nach der Intervention zeigt sich in allen Subskalen und dem Gesamtwert ein Anstieg, wobei der Anstieg bei 5 von 7 Punktwerten signifikant war. Unklar bleibt an dieser Stelle, welche praktische Relevanz dieser signifikante Anstieg hat. Darüber hinaus zeigte die RoH-Intervention besonders bei Betreuungskräften Veränderungen v. a. im Vergleich zu Pflegefachpersonen und Pflegeassistent*innen, was auf einen Vorteil niedrigschwelliger, interaktiver Trainingsmethoden hinweist. Möglicherweise führen die unterschiedlichen Aufgaben in der täglichen Pflegepraxis zu einer unterschiedlichen Aufmerksamkeit. Besonders bei Betreuungskräften zeigten sich deutliche Haltungsveränderungen. Möglicherweise liegt hier ein höheres Lernpotenzial, da Bewohner*innensicherheit in ihrer Qualifizierung bisher kaum thematisiert wird. Diese Annahme sollte in künftigen Studien durch eine differenzierte Erfassung des Vorwissens geprüft werden. Einschränkend muss erwähnt werden, dass die RoH-Intervention ohne Vergleichsgruppe oder einer weiteren Intervention abgesichert wurde. Somit ist unklar, ob die gefundenen Effekte tatsächlich auf die RoH-Intervention oder auf die Tatsache, dass überhaupt eine Intervention durchgeführt wurde, zurückzuführen sind. Auch bleibt unklar, ob die gemessene veränderte Haltung auch zu einer tatsächlich veränderten Arbeitsweise führt. Dazu zählen etwa eine erhöhte Risikowahrnehmung, häufigeres Ansprechen sicherheitsrelevanter Beobachtungen oder eine konsequentere Anwendung von Hygienestandards. Solche Verhaltensveränderungen wurden in dieser Studie jedoch nicht objektiv erfasst. Beide Punkte sollten in nachfolgenden Projekten adressiert werden.

Unabhängig von dieser Einschränkung bei der Interpretation der Ergebnisse, empfehlen die Teilnehmenden in hohem Maße die Intervention. Obwohl die Weiterempfehlungsrate unter der in der Studie von Zimmermann et al. (97,8 %) [10] liegt, kann geschlussfolgert werden, dass diese Maßnahme in der Praxis Anklang gefunden hat, was ebenfalls für eine dauerhaften Etablierung der Intervention spricht. Zukünftig könnten auch spezifischere RoH-Interventionen (beispielsweise speziell für Medikamentenmanagement oder Mobilisation) entwickelt werden.

Insgesamt zeigt die vorliegende Arbeit, dass RoH-Interventionen die Haltung von Mitarbeitenden der Langzeitpflege hinsichtlich der Bewohner*innensicherheit fördern und somit das Bewusstsein für Risiken und Fehlerquellen erhöhen können. Es bleibt jedoch ungewiss, ob die positiven Effekte langfristig und nachhaltig sind.

### Limitationen

Trotz der gewonnenen Erkenntnisse sind einige Limitationen zu beachten. Der G‑APSQshort wurde weder validiert noch sprachlich an die heterogene Zielgruppe angepasst, was zu Verständnisschwierigkeiten führte und teils Erklärungen durch die Forschenden nötig machte. Das Antwortverhalten könnte dadurch sowie durch soziale Erwünschtheit beeinflusst worden sein. Zudem ist nicht auszuschließen, dass Gespräche über den RoH nach der ersten Intervention die Fehlererkennung oder die Haltung zur Bewohner*innensicherheit verändert haben. Die Analysen basieren auf Mittelwerten; individuelle Auswertungen könnten abweichen. Auch Gruppeneffekte wie Rollenverteilungen oder Hierarchien könnten die Ergebnisse beeinflusst haben.

## Fazit für die Praxis

- RoH-Interventionen sind ein niedrigschwelliges und innovatives Instrument im Qualitätsmanagement der Langzeitpflege und fördern die Haltung der Mitarbeitenden für die Bewohner*innensicherheit.
- Die Teilnahme von multiprofessionellen Teams stärkt das Verständnis für unterschiedliche Rollen und Aufgaben und betont die Bedeutung jedes Teammitglieds bei der Identifikation von Sicherheitsrisiken.
- RoH-Szenarien sind an die Bedürfnisse von Pflegeeinrichtungen anpassbar und helfen, Kompetenzdefizite zu identifizieren. Diese Defizite können durch gezielte Schulungsmaßnahmen adressiert werden, was zu einer nachhaltigen Verbesserung der Pflegequalität führt.

## Data Availability

Die erhobenen Datensätze können auf begründete Anfrage beim korrespondierenden Autor angefordert werden.
